# What Happens Inside the Germinating Grain After Microbial Decontamination by Pulsed Electric Field? Data-Driven Multi-Omics Helps Find the Answer

**DOI:** 10.3390/molecules30040924

**Published:** 2025-02-17

**Authors:** Milena Stranska, Adam Behner, Jaroslava Ovesna, Pavel Svoboda, Jana Hajslova

**Affiliations:** 1 Department of Food Analysis and Nutrition, University of Chemistry and Technology, Technicka 3, 166 28 Prague, Czech Republic; adam.behner@vscht.cz (A.B.); jana.hajslova@vscht.cz (J.H.); 2 Crop Research Institute in Prague, Drnovska 507/73, 161 06 Prague, Czech Republic; ovesna@vurv.cz (J.O.); pavel.svoboda@vurv.cz (P.S.)

**Keywords:** pulsed electric field, barley, *Fusarium* micromycetes, abiotic stress, metabolomics, transcriptomics, multi-omics

## Abstract

Pulsed electric field (PEF) has previously been recognized as a method of gentle food processing, and its use has been shown to be helpful in reducing the levels of toxigenic *Fusarium* micromycetes developed during malting. The aim of this study was to describe the effects of PEF on gene expression and metabolite production at the pre-finishing stage of barley malting by using a novel multi-omics data-driven approach. The study helps to uncover the processes occurring in the germinated grain and discusses the up-/downregulation of genes and metabolites in relation to fungal infection and/or PEF-induced abiotic stress. Among the factors upregulated by PEF and previously described as supportive against *Fusarium* diseases, we identified the increased expression of genes encoding vegetative gp1-like protein, which positively correlated with flavonoids, (methylsulfanyl)prop-2-enoates, triterpenoid glycosides, and indole alkaloids. On the other hand, some genes associated with barley resistance to fungal infection were also overexpressed in the untreated control (in particular, genes encoding ethylene response factor 3-like, putrescine hydroxycinnamoyltransferase 3-like, and dirigent protein 21-like). This study provides the first ‘data-driven’ basic research results that contribute to the understanding of the role of PEF as an effective fungal decontamination strategy and allows the formulation of new hypotheses related to *Fusarium* pathogen crosstalk.

## 1. Introduction

The research on plant metabolic pathways has advanced significantly in recent decades, driven by breakthroughs in genomic, transcriptomic, proteomic, and metabolomic studies. These comprehensive investigations have yielded vast amounts of information on the biochemical and physiological processes in plants and microorganisms. The genome sequencing of numerous plant species has facilitated the identification of genes encoding key enzymes involved in essential biosynthetic pathways. Meanwhile, the rise of metabolomics, powered by cutting-edge analytical techniques, has enabled the detailed analysis of a broad spectrum of plant metabolites.

A wide array of bioinformatic tools and databases such as KEGG [1], YMDB [2], PlantCyc [3], MetaCyc [4], GNPS [5], and Cytoscape [6], among others, have been developed to help scientists explore and understand the intricate biochemical pathways in plant organisms [7,8]. However, despite the wealth of data available in these resources, challenges remain in covering specific aspects of secondary metabolism, particularly in relation to responses to abiotic stresses (such as drought, salinity, or extreme temperatures) or biotic stresses caused by pathogenic microbial interactions [8]. These databases often fall short in providing clear and definitive answers to these complex questions.

The complexity of the plant metabolome [9,10], coupled with the insufficiently mapped knowledge databases, has been a topic of ongoing discussion [11]. This lack of understanding often hinders the accurate interpretation of metabolomic studies, which are crucial for improving agricultural practices, such as crop cultivation and plant resistance, together with minimizing fungal diseases, as well as for improving the processing technologies of plant-based foods and minimizing the occurrence of undesirable fungal metabolites. One of the food processing technologies particularly susceptible to the spread of microbial infections, especially toxinogenic micromycetes of the *Fusarium* genus, is barley malting. During the malting process, the barley is soaked and germinated for several days under humid conditions, which create an environment conducive to the spread of fungal pathogens naturally present on the grain surface, followed by toxin production [12,13,14]. However, as demonstrated in recent studies, treating the barley grains with short, intensive electric pulses (food processing technology called pulsed electric field, PEF) prior to malting disrupts the viability of these fungi through electroporation, significantly reducing the spread of fungal pathogens [15,16,17]. Tackling the issue of minimizing the development of *Fusarium* micromycetes during malting using PEF holds great potential. Consequently, it is crucial to investigate how this intervention influences the physiology and metabolism of germinating grains, as well as the interactions between the grain and the pathogen.

Although some information on the mutual interaction between plants and *Fusarium* species is available, most studies have been conducted on cereals under field cultivation conditions [18,19,20]. Field or greenhouse experiments have shown, for instance, that metabolites closely associated with pathogenesis, which are upregulated in cereal varieties resistant to *Fusarium* diseases, include phenylpropanoids, flavonoids, terpenoids, hydroxycinnamic acid amides, and lignans [18,19,20,21]. Similarly, at the genomic and transcriptomic levels, numerous pathogenesis-related genes involved in the development of field *Fusarium* diseases have been identified [21]. There are also publications utilizing metabolomics and other “omics” disciplines to investigate the effects of biotic and abiotic stress on plant physiology [22,23]. However, the processes occurring during barley growth in the field may differ significantly from those taking place during barley germination in malt houses. To the best of our knowledge, no studies currently exist that explore biochemical changes in barley (or other cereals) germinated under malting or similar food processing technologies and their relationship with *Fusarium* pathogens and/or abiotic stress. This study fills this knowledge gap by providing novel insights into gene expression and metabolic changes in germinated barley subjected to PEF intervention. Using a combined metabolomics–transcriptomics approach, it contextualizes these findings within the framework of *Fusarium* diseases.

## 2. Results and Discussion

### 2.1. Analysis of Fusarium Fungi

**Figure 1:**
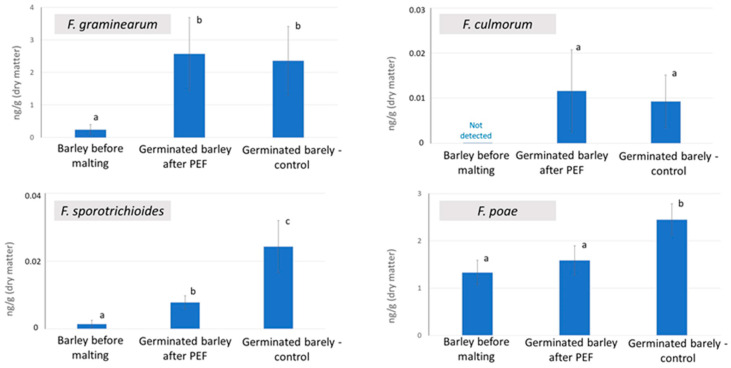
Concentration of fungal DNA in samples of barley and malted barley; the error bars represent the standard deviation, *n* = 9. Data were statistically processed using a two-sample *t*-test with unequal variance; statistical differences (*p*-value < 0.05) are indicated by letters.

In this study, malting barley grown in the field under the standard agricultural practice was used. The initial analysis revealed natural infection by *Fusarium* fungi in the grains at low levels, with specific concentrations measured as follows: 0.2423 ± 0.1524 ng/g for *F. graminearum*, 0.0014 ± 0.0011 ng/g for *F. sporotrichioides*, and 1.3307 ± 0.2613 ng/g for *F. poae*. After 72 h of germination, with and without the PEF pre-treatment, the barley samples were analyzed using RT-PCR to quantify pathogen DNA content. As shown in Figure 1, despite the fact that the fungal DNA content increased during germination for all species studied, the content of *F. poae* and *F. sporotrichioides* DNA was significantly lower in the germinated barley samples treated with PEF compared to the untreated control. In contrast, the DNA levels of *F. culmorum* and *F. graminearum* remained relatively unchanged. Although a slight increase in mean DNA concentration for these two species was observed following PEF treatment, the changes were not statistically significant. These trends align with those observed by Karabin et al. and Prusova et al., who conducted similar experiments using barley artificially inoculated with *Fusarium* species [17,24].

### 2.2. Multi-Omics Analysis and Data Processing

The level of expressed genes in the germinated barley, with and without prior PEF treatment, was analyzed by the next-generation sequencing (NGS), and changes in metabolite production were recorded by ultra-high-performance liquid chromatography coupled with high-resolution mass spectrometry (U-HPLC-HRMS/MS). As mentioned in the Introduction, although significant advances have been made in metabolomics and transcriptomics in plant research in recent years, there are still significant knowledge gaps, particularly in understanding plant-pathogen crosstalk. This is primarily due to the inherent complexity of plant and microbial metabolism and the still-incomplete mapping of these processes. Such limitations significantly hinder the application of the so-called “knowledge-driven” approach to data processing, which relies on integrating information from databases and libraries and establishing cross-connections across various levels of omics disciplines [25]. Unlike humans and experimental mammals, whose metabolism is comparatively well studied and described, plants and fungi possess unique and highly complex biosynthetic pathways. These pathways are not only species-specific but often vary significantly even between different varieties of the same species. The influence of biotic or abiotic stress adds further layers of complexity, introducing additional unknowns into this area of research.

**Figure 2:**
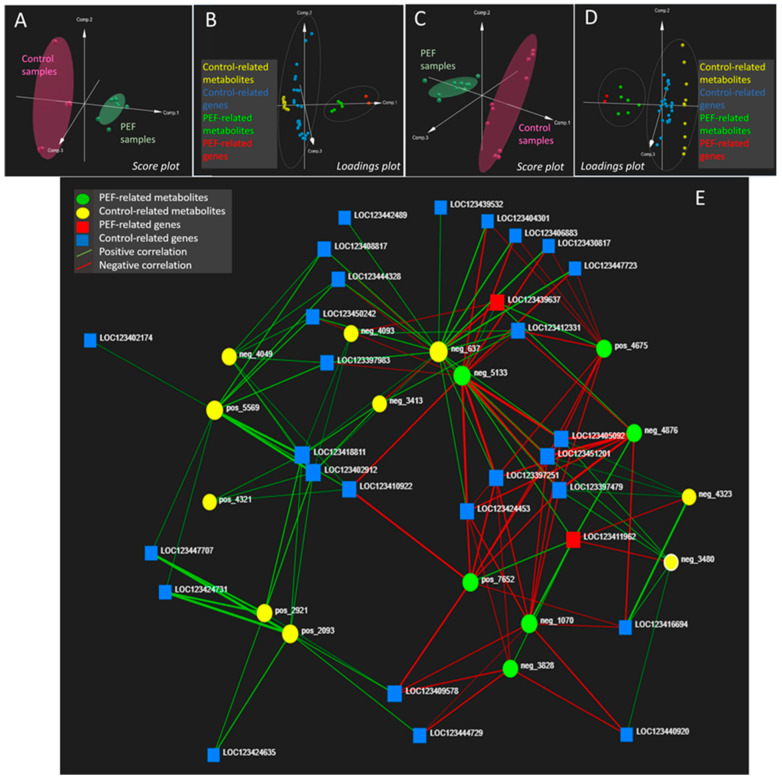
(**A**) Score plot of Multiple Co-Inertia Analysis (MCIA); (**B**) loading plot of Multiple Co-Inertia Analysis (MCIA); (**C**) score plot of Data Integration Analysis for Biomarker discovery using Latent cOmponents (DIABLO) analysis; (**D**) loadings plot of Data Integration Analysis for Biomarker discovery using Latent cOmponents (DIABLO) analysis; (**E**) transcriptomics–metabolomics correlation network; correlated components with correlation coefficient value ≥ 0.8.

Following that, for data processing in this study, a new multi-omics approach utilizing the ‘data-driven’ correlation method described by Ewald et al. [25] was employed. This approach involved an initial selection of statistically significant variables from each single-omics level prior to the correlation analysis. Unlike the original protocol by Ewald et al. [25], the pre-selection of statistically significant variables (as described in Section 3.8, Variable Filtration at the Single-Omics Level, of Section 3) was implemented to limit the number of variables entering the correlation analysis and to filter out correlations with low significance. The complete lists of output features from the transcriptomics and metabolomics analyses are provided in Table 1 and Table 2, respectively, with all relevant details about the identified genes and metabolites available in the Supplementary Materials (MS Excel file). These output lists were subsequently correlated using the OmicsAnalyst software (v. 2.0), following the methodology of Ewald et al. [25]. The results of the data-driven correlation between transcriptomics and metabolomics data are presented in Figure 2.

For better clarity, all positive and negative correlation relationships illustrated by a relatively uncluttered ‘multi-omics spider’ are also listed in the Supplementary Materials (MS Excel file). As shown in Table 1 and Figure 2, the only two barley genes whose expression was upregulated by PEF were the genes encoding vegetative gp1-like protein (gene ID LOC123411962), a late embryogenesis abundant protein (LEA) EMB564-like (gene ID LOC123439637). The vegetative gp1-like protein is known as a part of the plant cell wall acting as a mechanical barrier against negative external influences, including the penetration of fungal pathogens [26]. The expression of the gene encoding this protein positively correlates with several metabolites, including amentoflavone 7,4′-dimethyl ether (met. ID neg_3828). According to the literature, flavonoids are known to protect plant cell wall integrity upon fungal infection by inhibiting the activity of specific cell wall degrading enzymes that are secreted by fungal pathogens to penetrate plant tissues [21,27]. There is also a positive correlation between the vegetative gp1-like protein gene overexpression and the upregulation of the compound from triterpenoid glycosides class, called (2,3,4-trihydroxy-5-{[11-hydroxy-1-(2-hydroxy-6-methylhept-5-en-2-yl)-3a,3b,6,6,9a-pentamethyl-hexadecahydro-1H-cyclopenta[a]phenanthren-7-yl]oxy}cyclohexyl)methyl acetate (met. ID pos_7652). Terpene glycosides generally belong to the plant secondary metabolites that act as protective factors against fungal pathogens [28,29], by inhibiting the fungal (1→3)-β-D-glucan synthase, a key enzyme for rigid fungal cell wall [30]. Another metabolite positively correlated with vegetative gp1-like protein gene expression was (2-methoxyphenyl)methyl 3-(methylsulfanyl)prop-2-enoate (met. ID neg_1070). Specifically, this compound itself has not been described in barley before, but related substances, derivatives of (methylsulfanyl)prop-2-enoate, have been identified as important components of the extract of other plants [31,32] for which antifungal activity has been demonstrated [32]. The above-described cluster of PEF-upregulated gene expression and metabolite production mutually correlated with coefficient ≥ 0.8 allows for the formulation of a hypothesis that PEF stimulates the biosynthesis of defensive biotic factors as a defense against *Fusarium* micromycetes, which could theoretically explain the reduction in the incidence of *Fusarium* species, specifically *F. sporotrichioides* and *F. poae* in PEF-treated barley (see Figure 1).

Another factor upregulated after the PEF treatment is the gene encoding the LEA protein EMB564-like. In plants, these hydrophilic LEA proteins are highly produced in embryos during the late embryogenesis stage and in vegetative organs in response to the plant tissue desiccation and drought stress conditions [33,34]. As can be seen in Figure 2E, the expression of the LEA-encoding gene positively correlates with the upregulation of metabolite 6,7-dimethoxy-N-methyl-2-[3-(1H-pyrrol-1-yl)-3-(thiophen-3-yl)propanoyl]-1,2,3,4-tetrahydroisoquinoline-3-carboxamide (met ID pos_4675), the isoquinoline alkaloid derivative. Despite the fact that this metabolite has not been described in any previous barley-associated study, its positive correlation with LEA protein production makes good sense in the context of the results of the study performed on callus plants [35], where the metabolites upregulated in response to drought stress were identified as intermediates of isoquinoline alkaloid biosynthetic pathways [35]. Given that drought stress cannot be taken into account during barley germination during malting (the usual relative humidity is between 90% and 100%), our data-driven correlation study suggests that the germinating grain accumulates LEA in response to another type of abiotic stress, most likely that caused by PEF treatment.

In the recent literature, there are many references cross-connecting the expression of LEA genes and plant hormones, e.g., abscisic acid (ABA), as one of the key players in the adaptive response to drought stress; under the stress conditions, the biosynthesis and accumulation of ABA is enhanced, which triggers LEA gene expression [36,37]. At the same time, it is known that ABA also triggers key defense mechanisms relevant to *Fusarium* infection [38,39]. Despite the fact that ABA was not among the metabolites identified in our study, we can indirectly generalize that the increased production of specific signaling molecules in reaction to PEF-induced abiotic stress triggers not only the accumulation of LEA but also the *Fusarium* defensive factors. This theory is supported by the PEF-induced upregulation of two metabolites found in our study, in particular 4,12-dihydroxy-6-(hydroxymethyl)-16-methyl-17-[(N’-methylcarbamimidamido)methyl]-2-oxa-25-azapentacyclo [22.3.1.1^3,7^.1^13,17^.0^9,20^]triaconta-3(30),4,6-trien-18-yn-10-yl acetate (met ID neg_4876), from the group of pentacyclic methylguanidine derivatives, and methyl 15-ethyl-10-[15-ethyl-18-(methoxycarbonyl)-17-methyl-10,17-diazatetracyclo [12.3.1.0^3,11^.0^4,9^]octadeca-3(11),4,6,8-tetraen-12-yl]-12-hydroxy-17-methyl-10,17-diazatetracyclo [12.3.1.0^3,11^.0^4,9^]octadeca-3(11),4,6,8-tetraene-18-carboxylate (met ID neg_5133), the derivative of indole alkaloids. As shown in previous studies, some of the polycyclic guanidine derivatives isolated from the natural resources show antifungal activities [40]. Simple polyamines then play a positive role in the response of plants to various stress conditions, including drought [41], and indole alkaloids have been previously described as substances enhancing tolerance of plants to drought- or *Fusarium*-induced stress [35,42,43]. Neither the methylguanidine derivative nor the indole alkaloid characterized in this study have been previously reported as constituents of barley, and their direct association with LEA has not been demonstrated in the literature before. Nevertheless, the relationships revealed by the data-driven multi-omics allow for the raising of new theories that need to be tested in the future and would never have emerged without the methodology used here.

On the other hand, several genes associated with barley’s ability to defend itself against fungal infection were overexpressed also in the PEF untreated (control) variant. One such gene is the gene encoding the ethylene response factor 3-like (ERF3) protein (gene ID LOC123416694), which induces the expression of pathogen-related proteins that help alleviate pathogen infection [44]; in addition to this role, proteins from the ERF family are involved also in plant growth and fruit maturation [45]. Another significant gene upregulated in untreated control is that encoding putrescine hydroxycinnamoyltransferase 3-like protein (gene ID LOC123405092), which belongs to the hydroxycinnamoyltransferase (HCT) family of enzymes. These enzymes catalyze the transfer of hydroxycinnamoyl groups in the biosynthesis of phenylpropanoids, from which monolignols and lignin, forming a mechanical barrier of the cell walls against infection, are further biosynthesized [46,47]. Similarly, another overexpression in the control variant was observed for the gene encoding the dirigent protein 21-like (gene ID LOC123451201), which has also been previously described as an important factor in relation to lignin biosynthesis in response to biotic and abiotic stress [48,49]. These trends may be related to the slightly lower levels of *F. culmorum* and *F. graminearum* in ‘control’ germinated barley without PEF intervention and illustrate well the complexity and ambiguity of the events occurring in a living plant organism.

The overexpression of all of these control-related genes positively correlates with the upregulation of some lipid species in control samples, in particular with lysophospholipid LysoPE(0:0/22:5(7Z,10Z,13Z,16Z,19Z)), metabolite ID neg_3480, and ceramide N-(1,3-dihydroxy-16-methyloctadec-4-en-2-yl)-2-hydroxyhexadecanamide, metabolite ID neg_4323, (see Figure 2E). In the current literature, the association of these metabolites with ERF has been described. The recent study by Wang et al. discusses the effects of ethylene and ERF on changes in the lipidome composition of peaches and presents the increased levels of ceramides and phospholipids [42], which is in agreement with our study. There are also studies in which the lipid profile was directly monitored in the context of resistance to *Fusarium* disease [50,51], and lysophospholipids have been identified as important signaling molecules involved in plant responses to stress [52].

The above discussion represents the interpretation of several correlations that explain well the ‘germinating grain-pathogen’ crosstalk and find support in the existing literature. At the same time, there are still many correlations that have not been elucidated in this study. It is evident that the multi-omics data-driven correlation of barley metabolomics and transcriptomics is very complicated, even though only a relatively small number of statistically significant variables from each omics layer entered the correlation analysis. Although some of the correlations make good sense, others cannot be confirmed on the basis of the available knowledge. The difficulty in interpreting complex biological relationships between expressed genes and metabolites is undoubtedly also related to the fact that correlated variables may not always be the end points of mutual interactions but may be the intermediates of various biosynthetic or signaling pathways that are not yet fully described. Although this study does not provide a comprehensive explanation of all the correlated relationships, it is undoubtedly useful material for any follow-up studies dealing with the issue of plant-pathogen crosstalk, even completely independent of the PEF technology, since all the positively correlated relationships in control-related genes and metabolites actually correspond to normal PEF-unaffected barley.

## 3. Materials and Methods

### 3.1. Experimental Material

Barley of cultivar Bojos, which is most commonly used for the production of Pilsner type of malt, was used as input material for the PEF experiments and follow-up malting. This barley was harvested in 2022, and its natural contamination by four *Fusarium* species (*F. graminearum*, *F. culmorum*, *F. sporotrichioides*, and *F. poae*) was determined by the below-described RT-PCR method.

### 3.2. PEF Treatment and Barley Malting

One kilogram of barley was pre-soaked for 10 min in 0.05 M phosphate buffer. After pre-soaking, the barley was exposed to PEF under 3.8 kV/cm voltage, 200 A current, 100 bipolar pulses, and 20 µs pulse width [17,24]. As a control, one kilogram of barley was pre-soaked in 0.05 M phosphate buffer without PEF treatment for the same time period and subjected to malting under the same conditions. The automatic micromalting system (RAVOZ^®^, Olomouc, Czech Republic), was used for barley steeping and germination. Both control and PEF-treated barley were malted according to following conditions: steeping for 48 h at 15 °C (8 h steeping; 12 h aeration break; 8 h steeping; 12 h aeration break; 4 h steeping; 4 h dripping), germination for 72 h at 15 °C; humidity 95–98%. After germination, the “green malt” samples from the PEF-treated and control variants were collected in the amount of 20 g, immediately snap-frozen in liquid nitrogen, and then stored at −180 °C until further analysis. The malting experiment was performed in three biological replicates, and from each replicate, the samples were taken in three repetitions.

### 3.3. Analysis of Fungal DNA

The DNA isolation and RT-PCR assay was performed as described by Prusova et al. [24]. In brief, the frozen samples were cryogenically homogenized, and DNA from 200 mg of each sample was isolated in duplicate using the Machery-Nagel (D) Plant II kit, with the follow-up addition of 10U of RNAse and proteinase K (Sigma-Aldrich, St. Louis, MO, USA). RT-PCR reactions were performed in 25 μL volumes consisting of 12.5 μL of TaqMan^®^ 2× Universal PCR MasterMix (ThermoFisher Scientific, Foster City, CA, USA), 300 nM of each of the forward and reverse primers (Generi Biotech, Hradec Kralove, Czech Republic), 200 nM of the probe (Generi Biotech, Hradec Kralove, Czech Republic), and 100 ng of template DNA and nuclease-free water (Sigma-Aldrich, St. Louis, MO, USA). Real-time primers and probes for the quantification of *Fusarium* species DNA are provided in Table S1 of the Supplementary Materials. Quantitative RT-PCR was performed in the QuantStudioTM 6 cycler (Thermo Fisher Scientific, Waltham, MA, USA). The DNA concentrations were finally recalculated to dry matter of germinated barley.

### 3.4. RNA Isolation for Transcriptomics

The samples were homogenized by using a cryogenic laboratory mill (IKA A11 basic, Verkon, Prague, Czech Republic). The RNA was extracted from 100 mg of frozen material using the TRIzol reagent (Invitrogen, Carlsbad, CA, USA) and purified with an RNeasy column in the presence of DNase (Qiagen, Hilden, Germany). The quality of the RNA was assessed using agarose gel electrophoresis and the Agilent 4200 TapeStation System (Agilent, Santa Clara, CA, USA). Each sample was represented by three independent replicates.

### 3.5. NGS Analysis and Data Treatment

To ensure the integrity and quality of the RNA samples for subsequent NGS analysis, a secondary check was performed at the company providing the NGS analysis (Seqme, Prague, Czech Republic). This step confirmed that the samples met the required standards. Next, sequencing libraries were prepared for each biological replicate of every sample, targeting the 3′ end of the transcripts. RNA-Seq libraries were prepared using the Lexogen QuantSeq 3′ mRNA-Seq Library Prep Kit FWD, incorporating Unique Molecular Identifiers (UMIs) for error correction and reducing amplification bias, along with dual indexing for accurate sample multiplexing. This approach targeted the 3′ ends of polyadenylated RNA, simplifying the transcriptome sequencing and enabling precise gene expression profiling. Library quality control (QC) was performed using the Agilent Bioanalyzer 2100 (Agilent, Santa Clara, CA, USA), Invitrogen Collibri Library Quantification Kit (Thermo Fisher Scientific Baltics, Vilnius, Lithuania), and Qubit 1X dsDNA High-Sensitivity Assay Kit (Thermo Fisher Scientific, Waltham, MA, USA) to assess size distribution, concentration, and integrity, ensuring high-quality libraries suitable for accurate sequencing with minimal technical variability. Subsequently, individual libraries were normalized to compensate for possible differences in RNA concentration and pooled for further analysis. The NGS analysis was performed using the Illumina NovaSeq platform with the following parameters: single-end sequencing, 50 base pairs per read, and a sequencing depth of 100 million reads. During the sequencing process, raw fastq files were generated for each sample. These files underwent data preprocessing to ensure quality and remove unwanted sequences. The initial quality assessment was conducted using the FastQC (v. 0.11.9) and multiQC (v. 1.12) programs. Trim Galore (v. 0.6.7) was then employed to eliminate low-quality reads and adapter sequences. The resulting data were again evaluated using FastQC and multiQC. After preprocessing, the reads were mapped to the respective genome reference (GCA_904849725.1) using Hisat2 (v. 2.2.1). To analyze the differential expression, the mapped reads were utilized. The count of mapped reads was obtained using featureCounts (v. 2.0.3) and stored in a count matrix. The normalization of read counts and identification of significantly expressed genes were performed using DESeq2 (v. 1.26.0). For further analysis, zeroes in the count matrix were replaced using “R” and routines within missMDA libraryR, which does not disturb the original data structure.

### 3.6. Isolation of Metabolites

All frozen samples were homogenized using a cryogenic laboratory mill (IKA A11 basic, IKA Works GmbH & Co. KG, Staufen im Breisgau, Germany). One gram of dry matter was weighed in to the 50 mL PTFE cuvette, and 10 mL of methanol was added. The cuvette with the homogenized sample was then placed on a laboratory shaker (HS 260 basic, IKA Works GmbH & Co. KG, Staufen im Breisgau, Germany) for 30 min (shaking at 240 RPM). The extract was then centrifuged (Rotina 35 R, Hettich Zentrifugen, Tuttlingen, Germany) for 2 min and 13,528 RCF (relative centrifugal force) and microfiltered through 0.2 µm spin filters (Ciro, Deerfield Beach, FL, USA). Finally, an aliquot of approx. 1 mL was transferred to a glass HPLC vial for further analysis by UHPLC-HRMS/MS. To eliminate potential system drift in the analytical system, the pooled extract (quality control sample (QC)) was prepared by mixing 100 µL of extract from each sample and analyzed. To exclude background signals from the laboratory processing of samples, a “processing blank” sample was prepared together with the analyzed samples.

### 3.7. Metabolomics Analysis

The UHPLC-HRMS/MS analysis was performed according to the study by Stranska et al. [11], with slight modifications. Briefly, for the separation of metabolites, the UHPLC system (Dionex UltiMate 3000 RS UHPLC system, Thermo Fisher Scientific, Waltham, MA, USA) used a reverse phase column of Acquity UPLC^®^ BEHC18 (100 mm × 2.1 mm; 1.7 m; Waters, Milford, MA, USA). The injection volume was 2 µL, the autosampler temperature was 10 °C, and the column temperature was 60 °C. The mobile phase consisted of (A) 5 mM ammonium in a mixture of milli-Q water:methanol (95:5, *v*/*v*) with 0.1% formic acid (*v*/*v*) and (B) 5 mM ammonium in a mixture of isopropanol:methanol:milli-Q water (65:30:5, *v*/*v*/*v*) with 0.1% formic acid (*v*/*v*). Methanol, isopropanol, ammonium formate, and formic acid (all of LC-MS grade) were obtained from Merck (Darmstadt, Germany). Deionized water (18 MΩ, 25 °C) was prepared using a Milli-Q^®^ system (Millipore, Bedford, MA, USA).

The SCIEX TripleTOF^®^ 6600 quadruple time-of-flight mass spectrometer (SCIEX, Concord, ON, Canada) was used for the detection in negative (ESI-) and positive (ESI+) ionization mode. To record the MS1 and MS/MS data, the TOF MS method (full scan) and the Information Dependent Acquisition (IDA) method were used. The working range was 100–1200 *m*/*z* for MS1 and 50–1000 *m*/*z* for MS/MS, and data were acquired between 0.5 and 19 min. The MS/MS spectra were collected for the eight most intensive ions of the MS spectra throughout the chromatographic run. The collision energy was 35 ± 15 V, and the QC sample was analyzed every ten samples during the sequence. Instrument control and data acquisition were operated with Analyst 1.7.1 TF software (SCIEX, Concord, ON, Canada), and qualitative analysis was performed using SCIEX OS software (v. 1.5.0.23389, SCIEX, Concord, ON, Canada).

For data mining and processing, the freely available MS-DIAL (v. 4.80)–MS-CleanR—MS-FINDER (v. 3.52) software platform was used according to the workflow published by Stranska et al. [53]. In the first step, the UHPLC-HRMS/MS data were processed and deconvoluted by MS-DIAL (v. 4.80, 2021, RIKEN, Tokyo, Japan). For peak picking, the MS1 and MS/MS tolerances were set to 0.03 and 0.1 Da in the centroid mode for both the datasets acquired in the ESI− and ESI+ mode. For peak alignment, the QC reference file was used with an RT tolerance of 0.05 min and mass tolerance of 0.015 Da. The minimum peak height for peak detection was set to amplitude 9000 (referring to 70% under the observed baseline for a blank injection). Zero values were replaced with 1/10 of the minimum peak height for all samples during the export of aligned results from MS-DIAL. In the second step, aligned results were cleaned with MS-CleanR (MetaToul-AgromiX Platform, Bordeaux, France). For data cleaning, all filters, including blank filters, a ghost peaks filter, incorrect mass, relative standard deviation, and relative mass defect were activated, with a minimum blank ratio set to 0.8, a maximum relative standard deviation (RSD) set to 30%, and a relative mass defect (RMD) ranging from 50 to 3000. The maximum RT difference and mass difference tolerance values were set to 0.025 min and 0.005 Da for Pearson correlation and ESI+/− data merging. In this step, feature clustering based on Pearson correlation and MS-DIAL peak character estimation algorithm (MS-DIAL-PCE) was implemented, and one peak (the most intense) was kept in each cluster. In the third step, the filtered features were automatically annotated with MS-FINDER (v. 3.52, 2021, RIKEN, Tokyo, Japan). The MS1 and MS/MS tolerances were set to 5 and 15 ppm, respectively. C, H, O, N, P, and S atoms were included for the formula finder parameter module. As a data source for automatic identification, the databases YMDB, ECMDB, PlantCyc, ChEBI, T3DB, NPA, KNApSAcK, LipidMaps, and PubChem were used. On the basis of the accurate mass and isotope ratio of MS1 ions, elemental compositions of candidate ions were proposed, and possible chemical structures of candidates were computed by comparing the experimental and in silico MS/MS spectra. Finally, MS-CleanR was launched, matched information from MS-FINDER automatic annotation with filtered features, and created a final data matrix with filtered and annotated features for further statistical analysis. In this step, MS-CleanR also matched data from the ESI+ and ESI− ionization mode into one data matrix.

### 3.8. Variable Filtration at the Single-Omics Level

Both data matrices from both single-omics levels were filtered using univariate statistical tools (Volcano plot—*t*-test, Fold change) according to Behner et al. [15], to exclude statistically insignificant features and prevent the overfitting of final models. Univariate methods were performed using the freely available web platform MetaboAnalyst (v. 5.0, Xia Lab, McGill, Montreal, QC, Canada), with *t*-test false discovery rate (FDR) adjusted *p*-value < 0.01 for metabolomics and <0.05 for transcriptomics and Fold change >2 for both single-omics. The general overview of feature reduction during data filtration for all data matrices is summarized in Tables S2 and S3 (MS Word file). The metabolomics data matrix was processed with MS Excel to perform the normalization of the data (total area sum normalization). In the next step, both metabolomics and transcriptomics data were separately uploaded to SIMCA software (v. 17.0, 2021, Sartorius, Göttingen, Germany), where multivariate Principal Component Analysis (PCA) and Orthogonal Partial Least Square Discriminant Analysis (OPLS-DA) were performed. Before the creation of every classification model, Pareto scaling and logarithmic transformation of the data were performed. The quality of the models was determined using the R2Y and Q2 validation parameters calculated by a 7-round internal cross-validation. Candidate biomarker compounds and statistically significant genes were selected based on OPLS-DA S-plots and Variable Importance of the Projection (VIP) plots together with Receiver Operating Characteristics (ROCs). Both filters were applied simultaneously in an orthogonal way, to assure proper statistical filtration and to keep only the strong biomarkers/genes relevant for further data interpretation. For selection, VIP scores greater than one (VIP score > 1) and ROC area under curve (AUC = 1) parameters were set.

### 3.9. Multi-Omics Data Integration

Multi-omics data integration was performed according to the workflow developed and described by Ewald et al. [25] based on the web-based freely available software OmicsAnalyst (v. 2.0, Xia Lab, McGill, Montreal, QC, Canada). Although each individual omics dataset (metabolomics + transcriptomics) was uploaded, the ‘Metadata table’ file, which included information about samples, groups, and factors, was also uploaded to the OmicsAnalyst interface. After uploading, auto scaling was applied to both datasets. For the first view of multi-omics data integration, Multiple Co-Inertia Analysis (MCIA) was performed in Dimensionality Reduction module. MCIA represents a robust method of finding related multidimensional components across multiple datasets. In the next step, Data Integration Analysis for Biomarker discovery using Latent cOmponents (DIABLO) was performed. This method is a generalized and supervised version of PLS (multiblock PLS-DA) that seeks to find related multidimensional components that maximally separate sample labels. In the next step, the correlation network based on the DIABLO feature selection method and the classical Spearman correlation analysis similarity matrix method was performed. For building a correlation network, only between-omics connections were considered. The corr. threshold for between-omics was set to >0.8.

## 4. Conclusions

This data-driven multi-omics study identified a number of significant factors (metabolites and expressed genes), the pattern of which was changed in response to the PEF treatment. The most important findings of this study are summarized as follows:

- In the PEF-treated samples, the upregulation of the gene encoding the vegetative gp1-like protein, previously described as barley protective factor against *Fusarium* disease and abiotic stress, has been found. At the same time, the upregulation of metabolites from the class of flavonoids, (methylsulfanyl)prop-2-enoates, triterpenoid glycosides, and indole alkaloids, previously described as important factors in the *Fusarium* pathogen defense, has been demonstrated. The PEF-induced upregulation of these genes/metabolites is well correlated with the apparent and statistically significant decrease in *F. sporotrichioides* and *F. poae* in germinated barley after the PEF treatment.
- On the other hand, the overexpression of some *Fusarium* defense factors was observed also in the control samples. These include the genes encoding ethylene response factor 3-like, putrescine hydroxycinnamoyltransferase 3-like, and dirigent protein 21-like. This trend may be related to the slight increase in the occurrence of *F. culmorum* and *F. graminearum* in germinated barley after the PEF intervention.
- It should be noted that when working with natural material, which naturally contains the representation of several *Fusarium* species, a clear interpretation of transcriptomic and metabolomic data in relation to the decrease/increase of fungal pathogens is very difficult. To confirm or refute the hypotheses raised on the basis of these pilot correlations and to correctly interpret the biochemical context, follow-up studies that include the variability of the input material (e.g., different barley varieties with different resistance/susceptibility to fungal diseases or different input levels of *Fusarium* pathogens, ideally single species) need to be performed. Nevertheless, the current study undoubtedly demonstrates the practical application of the recently published protocol of data-driven multi-omics and has provided the first important results on which future follow-up research can be built.

## Figures and Tables

**Table 1:** List of expressed genes selected at the single-omics transcriptomics level.

Gene No.	PEF-Induced Upregulation/Downregulation	Gene ID	Gene Interpretation
1	PEF-related	LOC123411962	vegetative cell wall protein gp1-like
2	PEF-related	LOC123439637	late embryogenesis abundant protein (LEA) EMB564-like
3	Control-related	LOC123440920	metallothionein like protein
4	Control-related	LOC123416694	ethylene response factor C3-like
5	Control-related	LOC123405092	putrescine hydroxycinnamoyltransferase 3-like
6	Control-related	LOC123451201	dirigent protein 21-like
7	Control-related	LOC123397479	small polypeptide DEVIL 5-like
8	Control-related	LOC123412331	metallothionein like protein
9	Control-related	LOC123418811	cytochrome b561 and DOMON domain containing protein
10	Control-related	LOC123402912	putative cell wall protein
11	Control-related	LOC123410922	codeine O-demethylase-like
12	Control-related	LOC123444328	subtilisin-like protease SBT 5.6
13	Control-related	LOC123397983	pectin esterase-like
14	Control-related	LOC123408817	skin secretory protein xP2-like
15	Control-related	LOC123450242	xyloglucan endotransglycosilase
16	Control-related	LOC123430817	3-ketoacyl-CoA synthase 12-like
17	Control-related	LOC123404301	phosphoribulokinase
18	Control-related	LOC123447723	calmodulin calcium-dependent NAD kinase
19	Control-related	LOC123406883	peroxidase 5-like
20	Control-related	LOC123442489	lecithin cholesterol acyltransferase 1-like
21	Control-related	LOC123439532	acyl transferase 7-like
22	Control-related	LOC123424453	indole-2 monooxygenase-like
23	Control-related	LOC123397251	protein BIG grain 1-like
24	Control-related	LOC123424731	peroxidase 2-like
25	Control-related	LOC123447707	photosystem II reaction center W protein
26	Control-related	LOC123424635	senescene associated gene 20-like
27	Control-related	LOC123444729	expansin A2
28	Control-related	LOC123409578	pathogenesis related protein 1-like
29	Control-related	LOC123402174	photosystem II 5 kDa protein, chloroplastic like

**Table 2:** List of metabolites selected at the single-omics metabolomics level.

Met. No.	Trend	Met. ID	Summary Formula	Systematic Name	Onthology
1	PEF-related	neg_3828	C32H22O10	(**1**)	flavonoid derivative
2	PEF-related	neg_1070	C12H14O3S	(**2**)	(methylsulfanyl)prop-2-enoate derivative
3	PEF-related	pos_7652	C39H66O8	(**3**)	triterpenoid glycoside derivative
4	PEF-related	pos_4675	C24H27N3O4S	(**4**)	tetrahydroisoquinoline derivative
5	PEF-related	neg_4876	C35H52N4O6	(**5**)	methylguanidine derivative
6	PEF-related	neg_5133	C42H54N4O5	(**6**)	indole derivative
7	Control-related	neg_3480	C27H46NO7P	(**7**)	phospholipide
8	Control-related	neg_4323	C35H69NO4	(**8**)	ceramide
9	Control-related	neg_4093	C35H63N2O3P	(**9**)	phosphorous acid derivative
10	Control-related	neg_4049	C32H56N6O4	(**10**)	N-acylpiperidines
11	Control-related	neg_3413	C27H46N6O4	(**11**)	piperidinecarboxamide derivative
12	Control-related	neg_637	C8H20N2O5	(**12**)	aminoalcohol
13	Control-related	pos_2093	C17H32O3	(**13**)	sesquiterpenoid derivative
14	Control-related	pos_2921	C19H28N2O4	(**14**)	phenylmethylamine derivative
15	Control-related	pos_4321	C43H68N8O10	(**15**)	cyclic depsipeptide derivative
16	Control-related	pos_5569	C34H53NO4	(**16**)	prostaglandin derivative

(**1**) amentoflavone 7,4′-dimethyl ether; (**2**) (2-methoxyphenyl)methyl 3-(methylsulfanyl)prop-2-enoate; (**3**) (2,3,4-trihydroxy-5-{[11-hydroxy-1-(2-hydroxy-6-methylhept-5-en-2-yl)-3a,3b,6,6,9a-pentamethyl-hexadecahydro-1H-cyclopenta[a]phenanthren-7-yl]oxy}cyclohexyl)methyl acetate; (**4**) 6,7-dimethoxy-N-methyl-2-[3-(1H-pyrrol-1-yl)-3-(thiophen-3-yl)propanoyl]-1,2,3,4-tetrahydroisoquinoline-3-carboxamide; (**5**) 4,12-dihydroxy-6-(hydroxymethyl)-16-methyl-17-[(N’-methylcarbamimidamido)methyl]-2-oxa-25-azapentacyclo [22.3.1.1^3,7^.1^13,17^.0^9,20^]triaconta-3(30),4,6-trien-18-yn-10-yl acetate; (**6**) methyl 15-ethyl-10-[15-ethyl-18-(methoxycarbonyl)-17-methyl-10,17-diazatetracyclo [12.3.1.0^3,11^.0^4,9^]octadeca-3(11),4,6,8-tetraen-12-yl]-12-hydroxy-17-methyl-10,17-diazatetracyclo [12.3.1.0^3,11^.0^4,9^]octadeca-3(11),4,6,8-tetraene-18-carboxylate; (**7**) LysoPE(0:0/22:5(7Z,10Z,13Z,16Z,19Z)); (**8**) N-(1,3-dihydroxynonadecan-2-yl)-2-hydroxyhexadec-3-enamide; (**9**) phosphorous acid (2,6-ditert-butyl-4-methyl-phenyl) bis(1,2,2,6,6-pentamethyl-3-piperidyl) ester; (**10**) 1-[4-[[4-[(1-hydroxy-2,2,6,6-tetramethyl-4-piperidyl)oxy]-6-(2,2,6,6-tetramethylpiperidino)-s-triazin-2-yl]oxy]-2,2,6,6-tetramethyl-piperidino]ethenone; (**11**) N2-(1-carbamoyl-2-cyclohexylethyl)-N1-cyclohexyl-4-(piperidine-4-carbonyl)piperazine-1,2-dicarboxamide; (**12**) octane-2,5-amino-1,3,5,7,8-pentaol; (**13**) 3,7,11-trimethyldodec-2-en-1-yl ethaneperoxoate; (**14**) N-[(3-hydroxy-4-{[(4-methoxyphenyl)methyl]amino}oxolan-2-yl)methyl]cyclopentanecarboxamide; (**15**) N-[20-(butan-2-yl)-17-[(4-methoxyphenyl)methyl]-9,13-dimethyl-7-(2-methylpropyl)-5,8,11,15,18,21-hexaoxo-10-(propan-2-yl)-icosahydro-1H-pyridazino [3,2-i]1-oxa-4,7,10,13,16-pentaazacyclononadecan-14-yl]-2-formamido-3-methylbutanamide; (**16**) N-benzyl-N-(2,2-dimethyloxan-4-yl)-7-[2-(3-hydroxyoct-1-en-1-yl)-5-oxocyclopentyl]heptanamide.

## Data Availability

The data presented in this study are available in Supplementary Materials.

## Supplementary Materials

The following supporting information can be downloaded at: https://www.mdpi.com/article/10.3390/molecules30040924/s1, Figure S1: The extracted ion chromatograms (XIC) (A), match of experimental vs. in silico MS/MS spectra with chemical structures of fragments colored green (B), and box plots before variable filtration (C) of PEF-related biomarkers; Figure S2: The extracted ion chromatograms (XIC) (A), match of experimental vs. in silico MS/MS spectra with chemical structures of fragments colored green (B), and box plots before variable filtration (C) of unique control-related biomarkers; Supplementary MS Excel file: Metabolites, genes, and correlations; Supplementary MS Word file: Table S1: Real-time primers and probes for the quantification of DNA of *Fusarium* species [54,55,56,57]. Table S2: The general overview of the feature reduction during data filtration for metabolomics data matrix. Table S3: The general overview of the feature reduction during data filtration for transcriptomics data matrix.
