# Supplementary material for: What Happens Inside the Germinating Grain After Microbial Decontamination by Pulsed Electric Field? Data-Driven Multi-Omics Helps Find the Answer
Source: Molecules. 2025 Feb 17;30(4):924. doi: 10.3390/molecules30040924 (PMC11858265; doi:10.3390/molecules30040924)
Supplement: Supplementary file 1 [file molecules-30-00924-s001.zip › Supplementary materials_tables_FINAL.pdf]

What happens inside the germinating grain after microbial decontamination by pulsed electric field? Data-driven multi-omics helps find the answer

Milena Stranska[1]*, Adam Behner[1], Jaroslava Ovesna[2], Pavel Svoboda[2] and Jana Hajslova[1]

[1] Department of Food Analysis and Nutrition, University of Chemistry and Technology, Prague, Technicka 3, 166 28 Prague, Czech Republic; adam.behner@vscht.cz (A.B.), nela.prusova@vscht.cz (N.P.), milena.stranska@vscht.cz (M.S.)

[2] Crop Research Institute in Prague, Drnovska 507/73, 161 06 Prague, Czech Republic; palicova@vurv.cz (J.P.), anna.tobolkova@gmail.com (A.T.)

*Correspondence: milena.stranska@vscht.cz, Tel. +420 220 443 142

Table S1: Real-time primers and probes for the quantification of DNA of *Fusarium* species.

Name		Sequence (5'→3') – fluorochrom/quencher	Reference
F. culmorum	**Forward**	TTCACTAGATCGTCCGGCAG	Leišová et al. (2006)
	**Reverse**	GAGCCCTCCAAGCGAGAAG	
	**Probe**	AAAGAAGTTGCAATGTTAGTG – VIC/MGB	
F. gramineum	**Forward**	CTCCGGATATGTTGCGTCAA	Yli-Mattila et al. (2008)
	**Reverse**	CGAAGCATATCCAGATCATCCA	
	**Probe**	TGAGAATGTCTTGAGGCAATGCGAACTTT – ABY/QSY	
F. sporotrichoides	**Forward**	GGTTGGCGTCTCACTTATAC	Köhl et al. (2015)
	**Reverse**	AATTTCTGATTCGCTAAAGTGG	
	**Probe**	CACACCCATAGTTACGTGTAA	
F. poae	**Forward**	GCTGAGGGTAAGCCGTCCTT	Yli-Mattila et al. (2008)
	**Reverse**	TCTGTCCCCCCTACCAAGCT	
	**Probe**	ATTTCCCCAACTTCGACTCTCCGAGGA – ABY/QSY	
***Fusarium* species (ITS)**	**Forward**	AACTCCCAAACCCCTGTGAACATA	Bluhm et al. (2004)
	**Reverse**	TTTAACGGCGTGGCCGC	
	**Probe**	CGCTCGAACAGGCATGCCCGCCAGAATAC – VIC/QSY	

Leišová, L., Kučera, L., Chrpová, J., Sýkorová, S., Šíp, V., & Ovesná, J. (2006). Quantification of Fusarium culmorum in Wheat and Barley Tissues Using Real-Time PCR in Comparison with DON Content. *Journal of Phytopathology, 154*(10). https://doi.org/10.1111/j.1439-0434.2006.01154.x

Yli-Mattila, T., Paavanen-Huhtala, S., Jestoi, M., Parikka, P., Hietaniemi, V., Gagkaeva, T., Sarlin, T., Haikara, A., Laaksonen, S., & Rizzo, A. (2008). Real-time PCR detection and quantification of Fusarium poae, F. graminearum, F. sporotrichioides and F. langsethiae in cereal grains in Finland and Russia. *Archives of Phytopathology and Plant Protection, 41*(4), 243-260. https://doi.org/10.1080/03235400600680659

Köhl, J., Lombaers, C., Moretti, A., Bandyopadhyay, R., Somma, S., & Kastelein, P. (2015). Analysis of microbial taxonomical groups present in maize stalks suppressive to colonization by toxigenic Fusarium spp.: A strategy for the identification of potential antagonists. *Biological Control, 83*, 20-28. https://doi.org/10.1016/j.biocontrol.2014.12.007

Bluhm, B. H., Cousin, M. A., & Woloshuk, C. P. (2004). Multiplex real-time PCR 436 detection of fumonisin producing and trichothecene-producing groups of Fusarium species. *Journal of Food Protection, 67*(3), 536-543. https://doi.org/10.4315/0362-028x-67.3.536

Table S2: The general overview of the features reduction during data filtration for metabolomics data matrix

Data treatment step	Processing	Filtration	Automatic identification	Statistical filter	
Software tool	MS-DIAL	MS-CleanR	MS-FINDER	Metaboanalyst: Volcano plot (p-value <0.01, FDR, FC>2)	VIP + ROC selection
Number of features	18428	1100	138	38	19

Table S3: The general overview of the features reduction during data filtration for transcriptomics data matrix

Data treatment step	Processing	Volcano plot (p-value <0.05, FDR, FC>2)	VIP + ROC selection
Number of reads	14890	524	29
