# Supplementary figures and images for: What Happens Inside the Germinating Grain After Microbial Decontamination by Pulsed Electric Field? Data-Driven Multi-Omics Helps Find the Answer
Source: Molecules. 2025 Feb 17;30(4):924. doi: 10.3390/molecules30040924 (PMC11858265; doi:10.3390/molecules30040924)

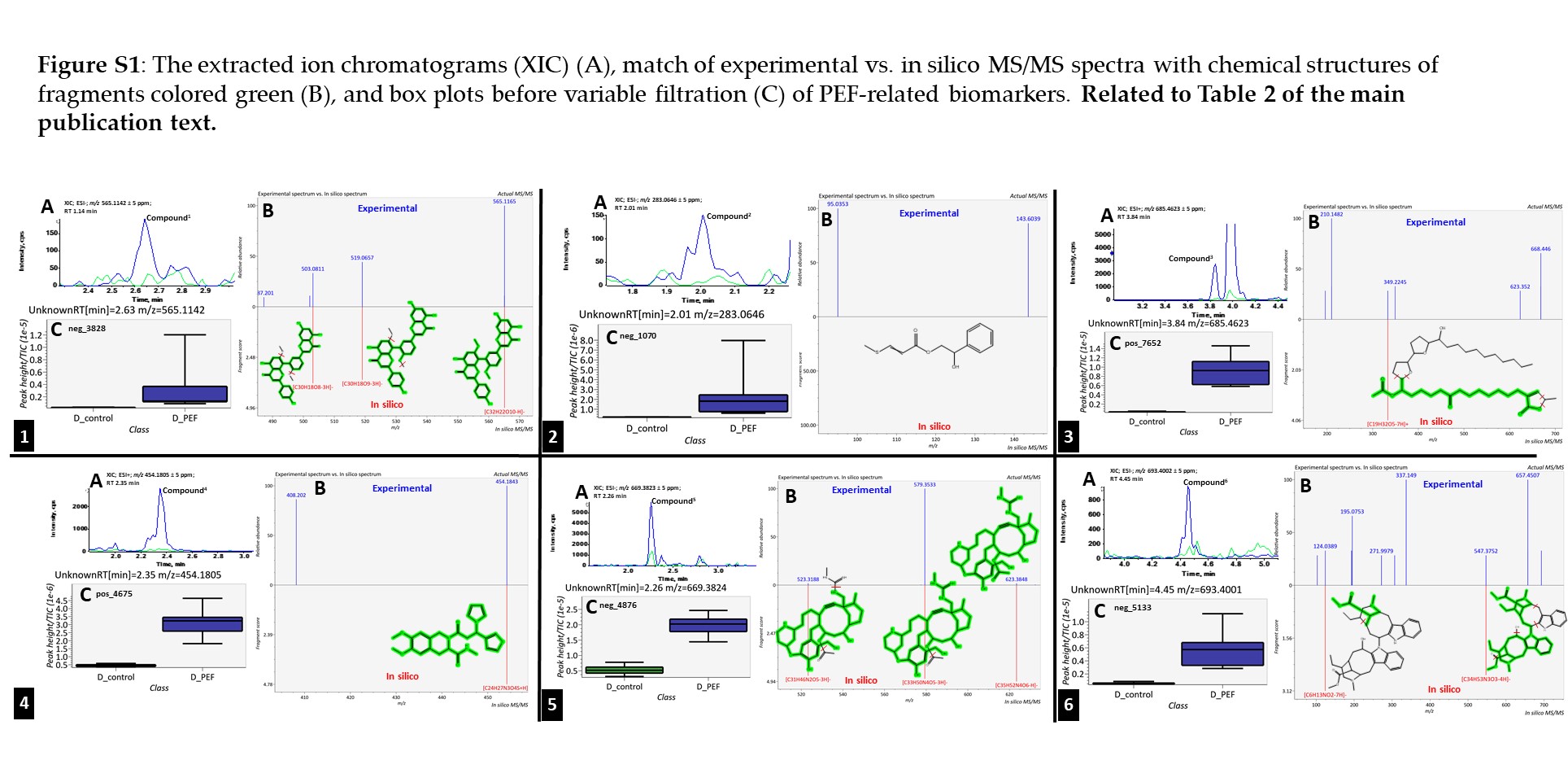

Supplement: Supplementary file 1 [file molecules-30-00924-s001.zip › Figure S1_FINAL_rev_proofs.jpg]

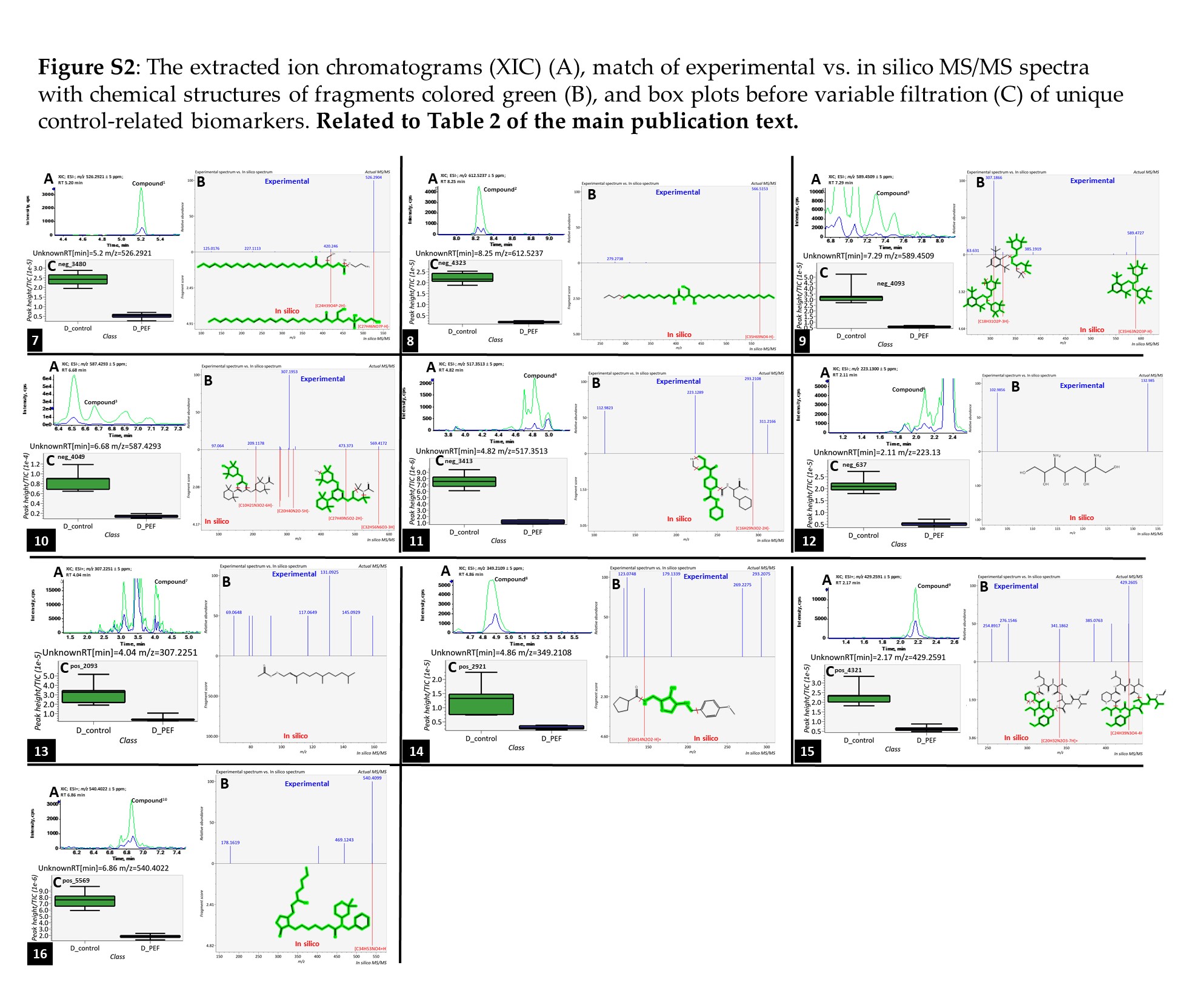

Supplement: Supplementary file 1 [file molecules-30-00924-s001.zip › Figure S2_FINAL_rev_proofs.jpg]
